# Supplementary figures and images for: DNA methylation clocks struggle to distinguish inflammaging from healthy aging, but feature rectification improves coherence and enhances detection of inflammaging
Source: GeroScience. 2025 Jan 18;47(3):3043–60. doi: 10.1007/s11357-024-01460-1 (PMC12181618; doi:10.1007/s11357-024-01460-1)

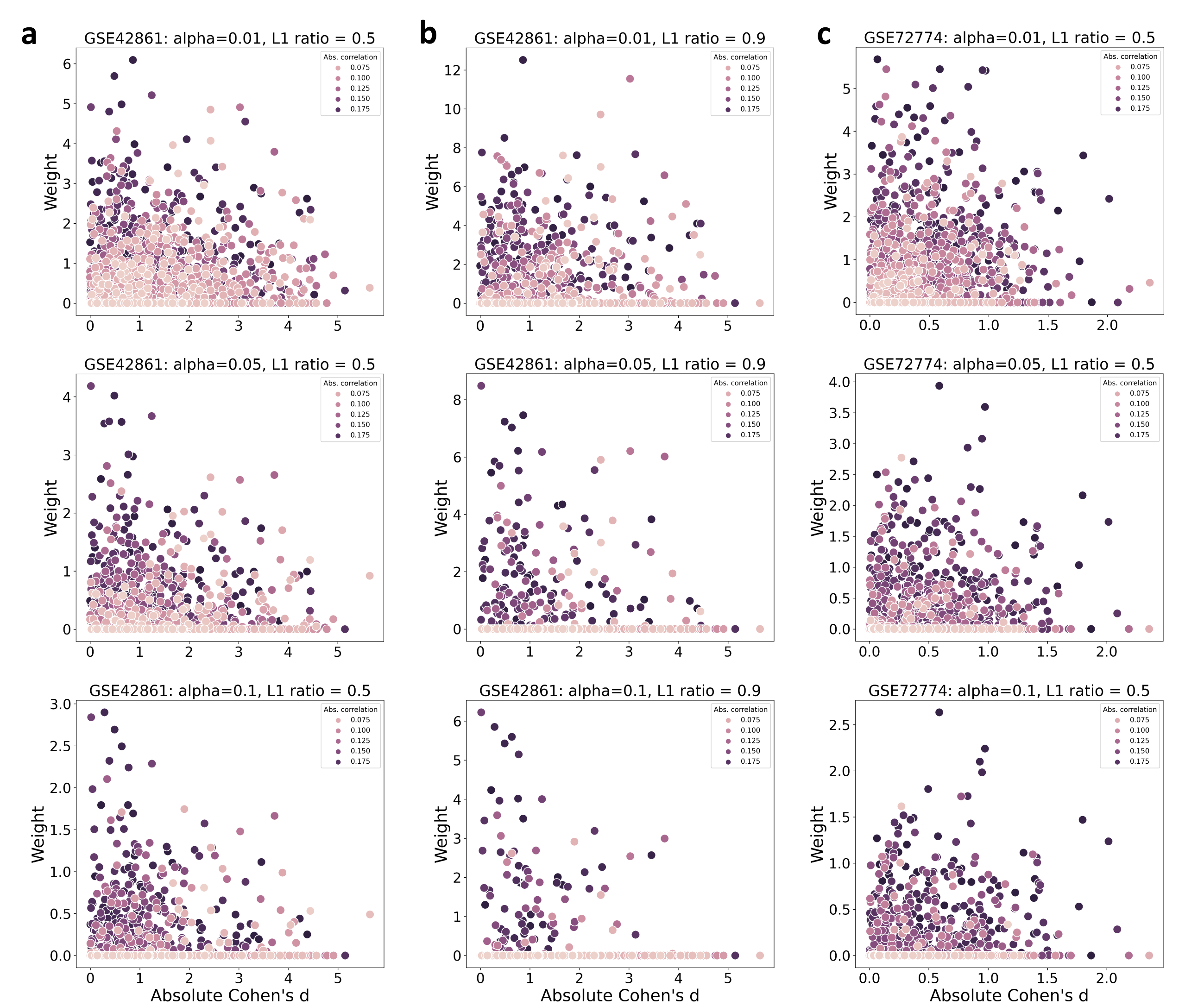

Supplement: Supplementary file 8 — Supplementary file8a GSE48261: learned EN weight vs. absolute value of effect size (Cohen’s d) with l1 ratio = 0.5 and (top) alpha = 0.01 (middle) alpha = 0.05 (bottom) alpha = 0.1. b GSE48261: EN weight vs. absolute value of effect size with l1 ratio = 0.9 and (top) alpha = 0.01 (middle) alpha = 0.05 (bottom) alpha = 0.1. c GSE72774: EN weight vs. absolute value of effect size with l1 ratio = 0.5 and (top) alpha = 0.01 (middle) alpha = 0.05 (bottom) alpha = 0.1 (TIF 18.3 MB) [file 11357_2024_1460_MOESM8_ESM.tif]

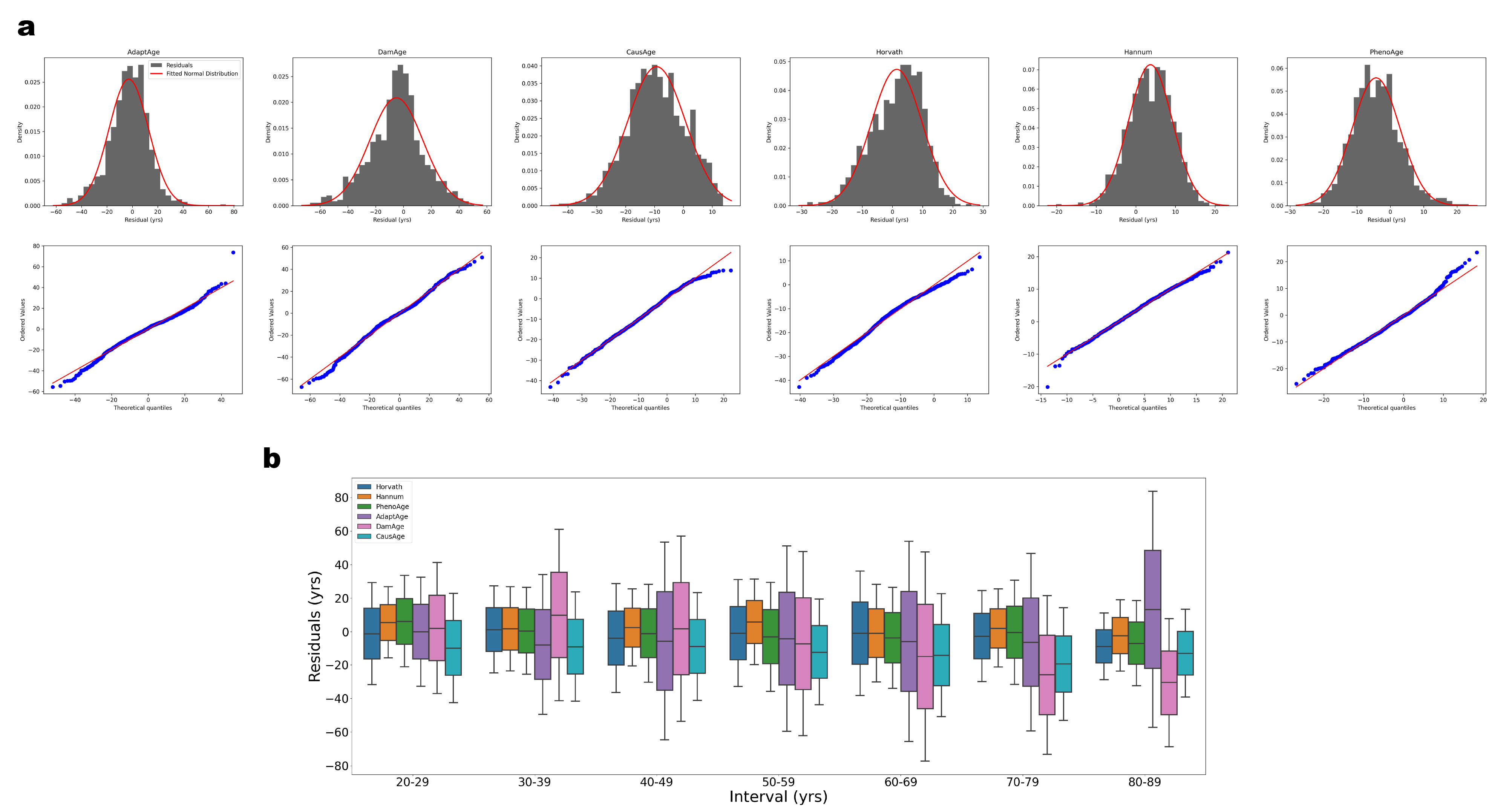

Supplement: Supplementary file 9 — Supplementary file9a (top) Histograms and fitted normal distributions of the residuals of the age predictions on the healthy cohort of the composite test dataset. (bottom) Q-Q plots for each model showing the goodness-of-fit of the normal distribution to the residuals. b 10-year age bins of the residuals for the healthy cohort of the composite test dataset for each of the tested models (PNG 348 KB) [file 11357_2024_1460_MOESM9_ESM.png]

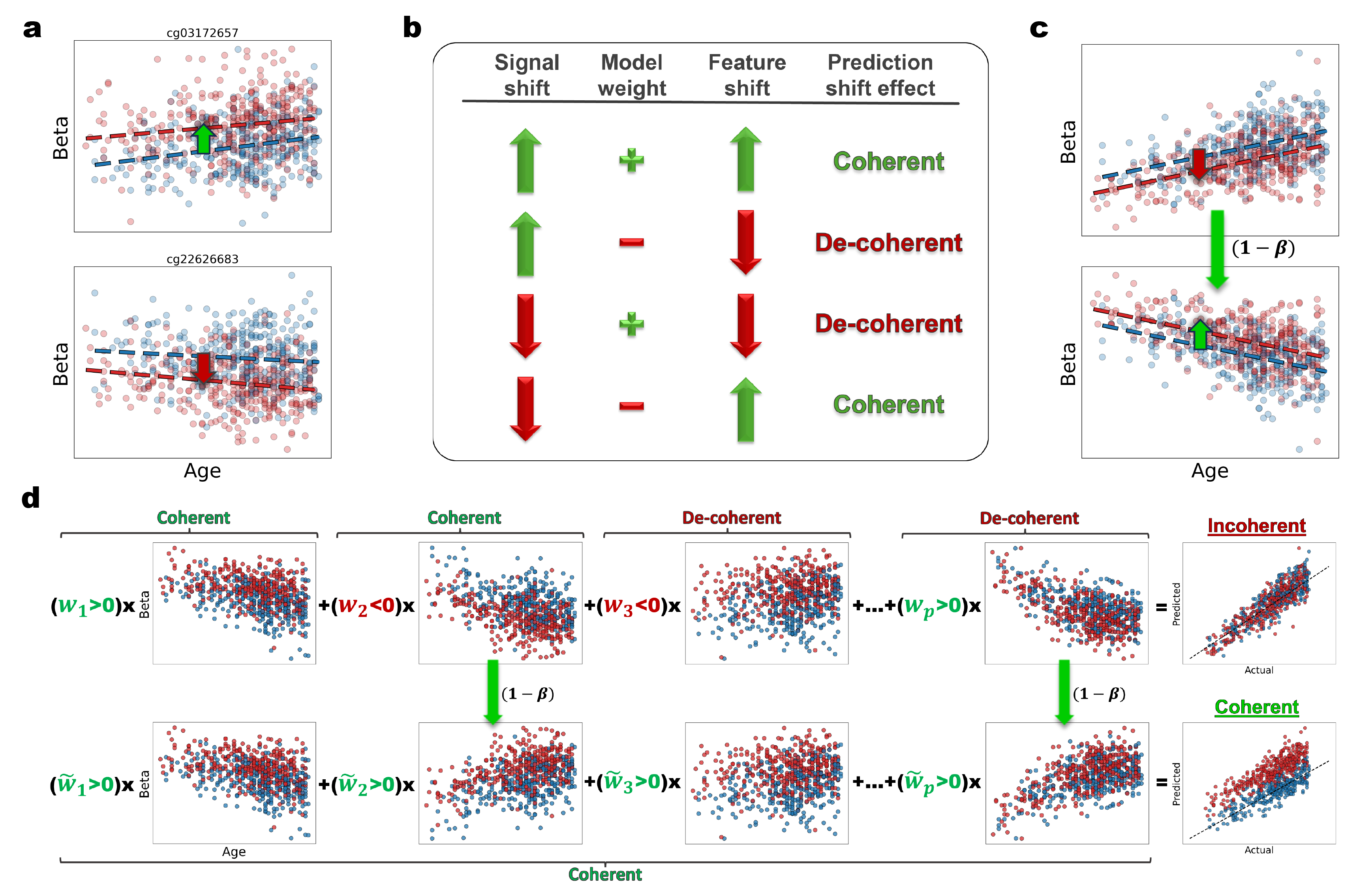

Supplement: Supplementary file 10 — Supplementary file10a Beta value versus age plotted for healthy controls (blue) and rheumatoid Arthritis patients (red) for representative CpGs in the GSE42861 dataset. (Top) A CpG for which the best-fit line for the RA cohort is shifted up from the HC cohort. (Bottom) A CpG for which the best-fit line for the RA cohort is down-shifted relative to the HC cohort. b Table summary of how the product of the weights and disease shifts for model probes combines to produce either coherent or de-coherent feature shifts. c Demonstration of the reflection transformation of the beta values for feature shift rectification (toy data). d (Top row) Visualization of how the linear combination (weights times beta values) of both coherent and de-coherent feature shifts produces an incoherent picture of biological age-acceleration in the presence of disease. (Bottom) Visualization of how a linear combination of the rectified feature shifts (positive weights, reflected beta values for CpGs downshifted for a disease) produce a coherent image of the biological age acceleration due to disease (PNG 1.86 MB) [file 11357_2024_1460_MOESM10_ESM.png]

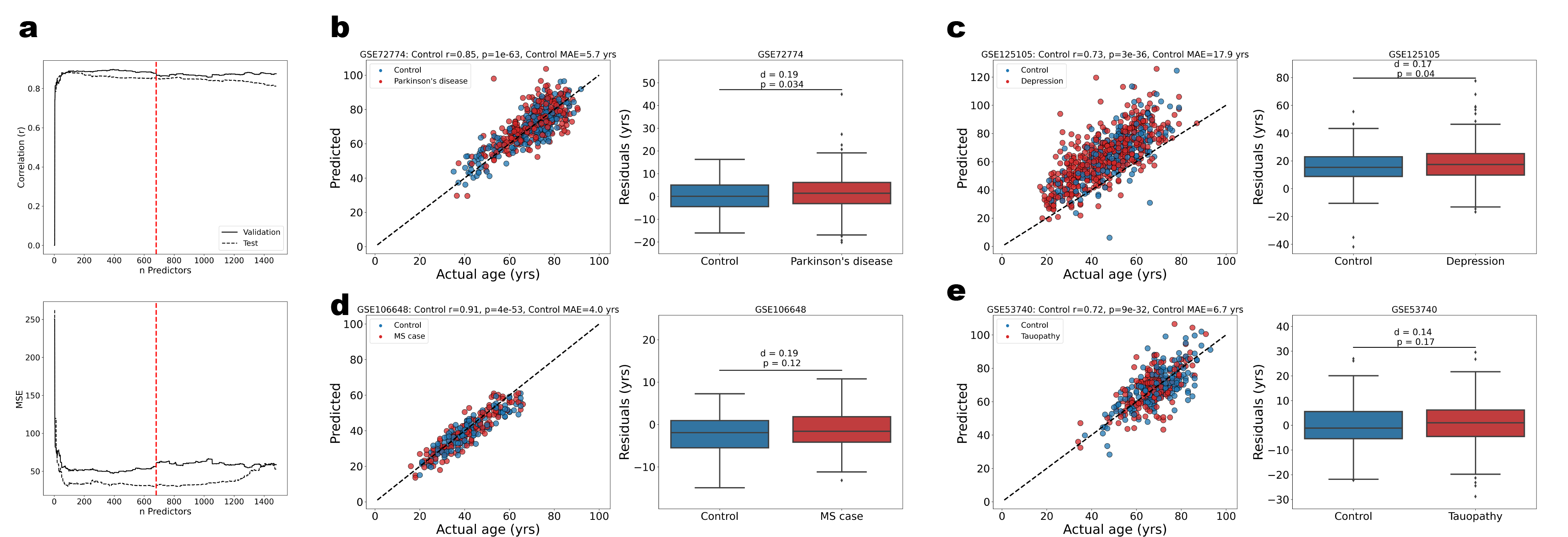

Supplement: Supplementary file 11 — Supplementary file11a (top) Correlation vs. number of features for RA coherent mFSS model trained on features ranked by age correlation. (bottom) Corresponding plot for MSE vs. number of features. Red dashed line indicates number of features which give the optimum validation MSE. b (Left) scatter plot for predicted age vs. actual age for GSE72774. (right) box plot of the residuals of the predictions. c (Left) scatter plot for predicted age vs. actual age for GSE125105. (right) box plot of the residuals of the predictions. d (Left) scatter plot for predicted age vs. actual age for GSE53740. (right) box plot of the residuals of the predictions. e (Left) scatter plot for predicted age vs. actual age for GSE53740. (right) box plot of the residuals of the predictions (PNG 616 KB) [file 11357_2024_1460_MOESM11_ESM.png]

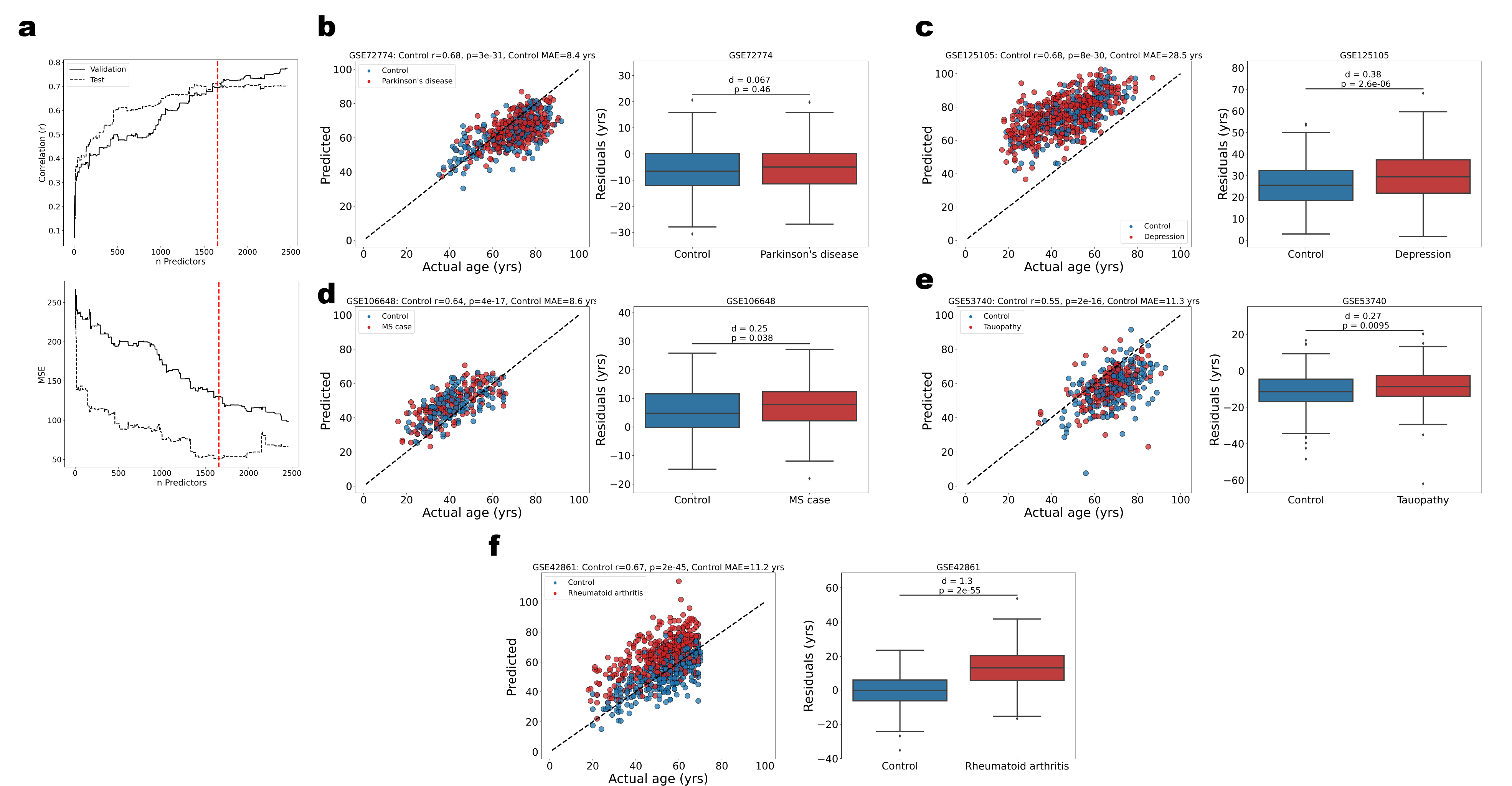

Supplement: Supplementary file 12 — Supplementary file12a (top) Correlation vs. number of features for RA-coherent mFSS model trained on features ranked by RA signal shift magnitude. (bottom) Corresponding plot for MSE vs. number of features. Red dashed line indicates number of features which give the optimum validation MSE. b (Left) scatter plot for predicted age vs. actual age for GSE72774. (right) box plot of the residuals of the predictions. c (Left) scatter plot for predicted age vs. actual age for GSE125105. (right) box plot of the residuals of the predictions. d (Left) scatter plot for predicted age vs. actual age for GSE106648. (right) box plot of the residuals of the predictions. e (Left) scatter plot for predicted age vs. actual age for GSE53740. (right) box plot of the residuals of the predictions. f (Left) scatter plot for predicted age vs. actual age for GSE42861. (right) box plot of the residuals of the predictions (PNG 827 KB) [file 11357_2024_1460_MOESM12_ESM.png]

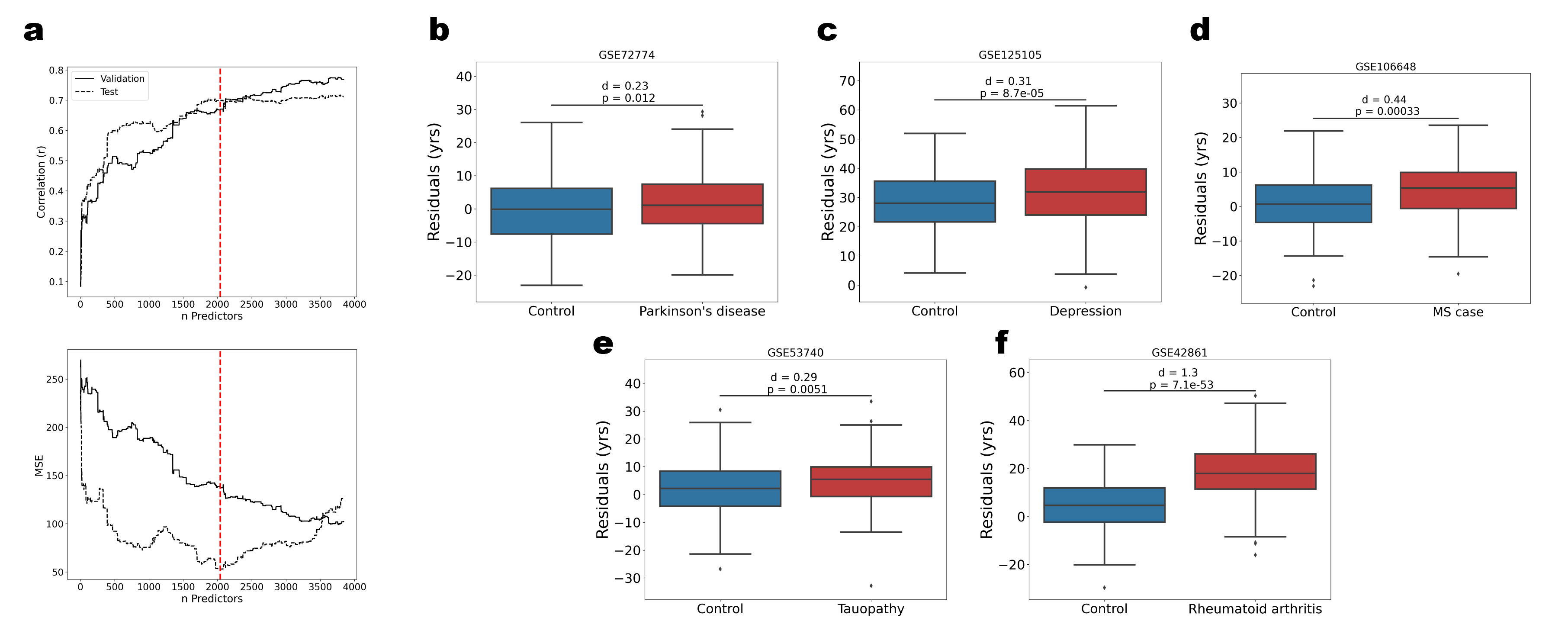

Supplement: Supplementary file 13 — Supplementary file13 a (top) Correlation vs. number of features for IF-coherent mFSS model trained on features ranked by IF signal shift magnitude. (bottom) Corresponding plot for MSE vs. number of features. Red dashed line indicates number of features which give the optimum validation MSE. b Residuals of the IR-mFSS model predictions on GSE72774 dataset comparing healthy controls (blue) to Parkinson’s disease patients (red). c Residuals of the IR-mFSS model predictions on GSE125105 dataset comparing healthy controls (blue) to multiple depression patients (red). d Residuals of the IR-mFSS model predictions on GSE10648 dataset comparing healthy controls (blue) to multiple sclerosis patients (red). e Residuals of the IR-mFSS model predictions on GSE53740 dataset comparing healthy controls (blue) to tauopathy patients (red). f Residuals of the IR-mFSS model predictions on GSE42861 dataset comparing healthy controls (blue) to rheumatoid arthritis patients (red) (PNG 249 KB) [file 11357_2024_1460_MOESM13_ESM.png]
